# Genetic underpinnings of recovery after stroke: an opportunity for gene discovery, risk stratification, and precision medicine

**DOI:** 10.1186/s13073-019-0671-5

**Published:** 2019-09-13

**Authors:** Julián N. Acosta, Stacy C. Brown, Guido J. Falcone

**Affiliations:** Division of Neurocritical Care and Emergency Neurology, Department of Neurology, Yale University School of Medicine, York Street, New Haven, CT 06510 USA

## Abstract

As the number of stroke survivors continues to increase, identification of therapeutic targets for stroke recovery has become a priority in stroke genomics research. The introduction of high-throughput genotyping technologies and novel analytical tools has significantly advanced our understanding of the genetic underpinnings of stroke recovery.

## Stroke: a growing epidemic

Stroke refers to a group of highly prevalent diseases of brain vasculature characterized by acute onset. Stroke is labeled as ischemic when a brain vessel is obstructed and the tissue distal to the obstruction undergoes necrosis. Hemorrhagic stroke occurs when a compromised vessel ruptures and blood extrudes into the brain parenchyma. In combination, these entities constitute the second leading cause of death and the third leading cause of disability worldwide [1]. Importantly, among young people (i.e., those ≤ 50 years of age) stroke constitutes the leading mechanism of long-term disability. This is an alarming finding considering that 10–15% of first-ever strokes occur in this population group. In addition, the absolute number of stroke survivors is increasing as the result of the continuous improvement of acute treatments and specialized care. These trends have energized research efforts oriented towards identifying novel biological mechanisms that are related to stroke recovery and utilizing these discoveries to develop new interventions and precision-medicine strategies for rehabilitation.

## Stroke genomics research: a collaborative endeavor

The field of population genetics offers powerful tools to advance these research avenues. Until the introduction of high-throughput genotyping technologies, genetic studies required a priori hypotheses and were driven by rare mutations that cause Mendelian conditions. In the past decade, the combination of new technological tools and the establishment of large research consortia, which are capable of obtaining and working with massive sample sizes, exponentially accelerated the pace of discovery of novel genetic risk loci for complex diseases such as stroke. In this setting, the identification of genetic polymorphisms that influence disease risk, drug effects, and even physiological characteristics grew exponentially, leading to the identification of ~ 100,000 polymorphism–disease associations, most of which are novel. Several of these newly discovered loci provide important physiological insights into disease biology and point to novel targets for drug development, risk stratification, and personalized medicine [2].

With the creation in 2008 of the International Stroke Genetics Consortium, a research network of more than 100 stroke genetics investigators from around the world, it became possible to harmonize the ascertainment of cases and controls, and to achieve sample sizes that were substantially larger than those utilized before. These advancements led to the completion of the largest genome-wide association study (GWAS) of ischemic stroke to date, an endeavor that identified more than 30 new loci for this disease [3]. These impactful results have galvanized stroke genetic research, identifying pathways that are related to different stroke subtypes and an overlap between stroke loci and related vascular traits (e.g., lipid levels, blood pressure, and atrial fibrillation). This led to follow-up studies that aimed to explore newly identified biological targets, developing prediction rules that are based on polygenic risk scores and characterizing differences in the genetic underpinnings of stroke in high-priority populations such as women and minorities, two groups at high risk of stroke that are underrepresented in genetic studies.

## Towards improving outcome and recovery after stroke

In recent years, functional recovery has received particular attention in stroke genomics research because of the increased survival and prevalence of the disease, and because it has been recognized that the prolonged post-stroke rehabilitation period provides an ideal opportunity to improve the overall outcome of stroke patients. The creation, within the International Stroke Genetics Consortium, of the Genetics of Ischemic Stroke Functional Outcome (GISCOME) network has been instrumental in advancing our understanding of how genetic variation influences outcome and recovery in ischemic stroke. This network has recently completed its first GWAS of clinical outcome after ischemic stroke [4]. The study was a meta-analysis of data from 6000 patients enrolled in 12 stroke genetic studies of outcome in Europe, the United States, and Australia, and it found a novel susceptibility locus for 3-month post-stroke functional outcome located on an intron of the gene LOC105372028 (top associated single nucleotide polymorphism (SNP) rs1842681). This locus is a previously reported trans-expression quantitative trait locus for PPP1R21, a gene encoding a regulatory subunit of protein phosphatase 1, which has been implicated in learning, memory, and neuronal plasticity in the brain. Furthermore, the study also found 29 suggestive (*p* < 10^− 5^) loci associated with functional outcome. The top SNPs in most of these loci are either associated with quantitative traits that affect the brain or are located within or near genes that are expressed in the brain. Of note, among the suggestive associations, three loci (PTCH1, TEK, and NTN4) are linked to genes that have been associated experimentally with a role in determining the amount of brain tissue that is irreversibly damaged by ischemia.

Another important initiative devoted to studying the genetic underpinnings of ischemic stroke recovery is the Genetic Contribution to Functional Outcome and Disability After Stroke (GODS) Project. This collaboration has also recently completed a well-designed first GWAS of stroke outcome and recovery [5]. The study evaluated data from 12 different studies involving 2482 stroke patients and identified another novel susceptibility risk locus (top associated SNP rs76221407) for worse outcome at 3 months. This risk locus appears with low frequency (2–3%) and encompasses *PATJ*, a gene that codes for Pals1-associated tight junction protein, which regulates multiple biological processes, including ion channel signaling and transport. No clear mechanism has yet been identified to explain the role of PATJ in stroke recovery, but follow-up functional studies are already underway. Of note, the identification of PATJ highlights the importance of studying low-frequency (minor allele frequency 1–5%) and rare (minor allele frequency < 1%) variants by using sequencing technologies coupled with appropriate statistical tools.

Important genetic discoveries have also been made for outcome and recovery after hemorrhagic stroke. In spontaneous intracerebral hemorrhage (ICH), genetic risk factors for poor functional outcome have been identified by evaluation of the volume of the hematoma upon admission to hospital, which is the most potent predictor of outcome in this condition [6]. For ICH located in the lobar regions of the brain, the epsilon 2 variant within apolipoprotein E (APOE), a well-studied risk factor for ICH via cerebral amyloid angiopathy, has been shown to increase both the volume of the hematoma and the risk of poor outcome [7]. For ICH compromising deep brain regions (thalamus, basal ganglia, brain stem, and cerebellum), a narrow genomic region at 17p12 (top SNP rs11655160) has been reported to associate with lower score on the Glasgow Coma Scale (a simple scale used to assess rapidly consciousness in critically ill neurological patients) at admission, larger hematoma volume, and poor 3-month post-bleed outcome [8]. Although the underlying mechanism is not yet clear, more than 30 copy-number variants are located in or around this locus and could be responsible for the observed clinical phenomena.

The Stroke, sTress, RehabilitatiON, and Genetics (STRONG) Study [9] constitutes a third crucial project studying the genetic basis of stroke outcome, recovery, and rehabilitation. While the networks described above aim to bring together different studies evaluating this specific area of research, STRONG is a genetic study that is specifically designed to identify prospectively, ascertain, and genotype stroke patients, evaluating rehabilitation outcomes in a standardized manner that includes in-person evaluations. The study is currently enrolling patients and its first results are expected soon.

## The importance of mechanistic research

The genetic association studies outlined above have yielded correlations between specific genes and outcome after ischemic or hemorrhagic stroke. In order to apply this knowledge in practice, we need to identify the biological pathways that mediate the observed allele–outcome associations. Significant advances are being made on this front too. As an example, a recent study evaluated the role of C-C chemokine receptor 5 (CCR5), which is implicated in learning, memory, and plasticity in hippocampal and cortical circuits, in stroke recovery [10]. The study utilized rodent models of stroke and traumatic brain injury to demonstrate that CCR5 knockdown reduces learning deficits and improves cognitive function, and that utilization of CCR5 antagonists promotes recovery in both conditions. The study also evaluated genetic epidemiology data from stroke patients, finding that naturally occurring loss-of-function mutations in CCR5 are associated with better motor recovery and decreased cognitive deficits several months after stroke. Importantly, CCR5 antagonist drugs are already available for use in clinical trials.

## Concluding remarks

In summary, functional outcome and recovery constitute important endpoints for genetic studies of stroke. The combination of improving statistical power and novel analytical tools will surely lead to the discovery of novel pathophysiological mechanisms underlying stroke recovery. Information on these newly discovered pathways can be used to develop new rehabilitation interventions and precision-medicine strategies aimed at improving management options for stroke survivors. The continuous growth and strengthening of existing dedicated collaborations and the utilization of standardized approaches to ascertain recovery-related phenotypes will be crucial for the success of this promising field.

## Abbreviations

CCR5: C-C chemokine receptor 5
GWAS: Genome-wide association study
ICH: Intracerebral hemorrhage
PATJ: Pals1-associated tight junction
SNP: Single nucleotide polymorphism
STRONG: Stroke, sTress, RehabilitatiON, and Genetics Study
